# 4-{[(4*Z*)-5-Oxo-2-phenyl-4,5-dihydro-1,3-oxazol-4-yl­idene]meth­yl}phenyl acetate

**DOI:** 10.1107/S1600536810014911

**Published:** 2010-04-28

**Authors:** Bharat B. Baldaniya, Mukesh M. Jotani, Edward R. T. Tiekink

**Affiliations:** a Department of Chemistry, M.G. Science Institute, Navrangpura, Ahmedabad, Gujarat 380 009, India; b Department of Physics, Bhavan’s Sheth R. A. College of Science, Ahmedabad, Gujarat 380 001, India; c Department of Chemistry, University of Malaya, 50603 Kuala Lumpur, Malaysia

## Abstract

The title mol­ecule, C_18_H_13_NO_4_, shows a dihedral angle between the terminal acetyl group (r.m.s. deviation = 0.0081 Å) and remaining non-H atoms (r.m.s. = 0.0734 Å) of 53.45 (7)°. The configuration about the central olefinic bond is *Z* and overall the mol­ecule has a U-shaped conformation. Supra­molecular chains along the *b*-axis direction are found in the crystal structure. These are stabilized by (C=O)⋯π(ring centroid of the 1,3-oxazole ring) inter­actions [3.370 (2) Å].

## Related literature

For background to the biological activity of 1,3-oxazole and imidazoles, see: Williams & Fu (2010 ▶); Khbnadidah *et al.* (2003 ▶). For related structures, see: Sun *et al.* (2007 ▶); Jotani & Baldaniya (2008 ▶).
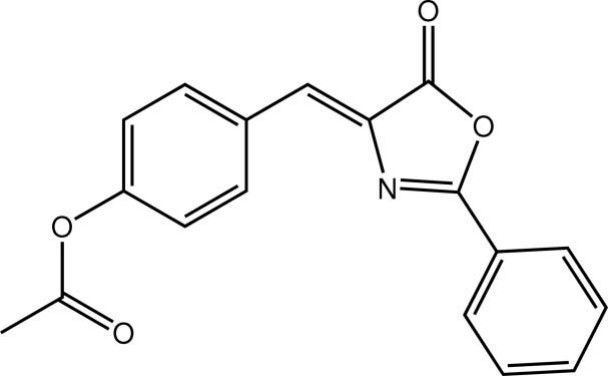


## Experimental

### 

#### Crystal data

C_18_H_13_NO_4_
*M* *_r_* = 307.29
Monoclinic, 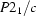

*a* = 13.3507 (15) Å
*b* = 3.9443 (9) Å
*c* = 28.527 (5) Å
β = 98.025 (11)°
*V* = 1487.5 (5) Å^3^
*Z* = 4
Cu *K*α radiation
μ = 0.81 mm^−1^
*T* = 293 K
0.40 × 0.20 × 0.15 mm

#### Data collection

Enraf–Nonius CAD-4 diffractometer
Absorption correction: ψ scan (North *et al.*, 1968 ▶) *T*_min_ = 0.852, *T*_max_ = 0.997
2593 measured reflections
2491 independent reflections
1795 reflections with *I* > 2σ(*I*)
*R*_int_ = 0.054
2 standard reflections every 3600 min  intensity decay: none

#### Refinement

*R*[*F* ^2^ > 2σ(*F* ^2^)] = 0.050
*wR*(*F* ^2^) = 0.140
*S* = 1.06
2491 reflections
210 parameters
H-atom parameters constrained
Δρ_max_ = 0.23 e Å^−3^
Δρ_min_ = −0.23 e Å^−3^

### 

Data collection: *XCAD4* (Harms & Wocadlo, 1996 ▶); cell refinement: *XCAD4*; data reduction: *XCAD4*; program(s) used to solve structure: *SHELXS97* (Sheldrick, 2008 ▶); program(s) used to refine structure: *SHELXL97* (Sheldrick, 2008 ▶); molecular graphics: *ORTEP-3* (Farrugia, 1997 ▶) and *DIAMOND* (Brandenburg, 2006 ▶); software used to prepare material for publication: *publCIF* (Westrip, 2010 ▶).

## Supplementary Material

Crystal structure: contains datablocks global, I. DOI: 10.1107/S1600536810014911/lh5032sup1.cif

Structure factors: contains datablocks I. DOI: 10.1107/S1600536810014911/lh5032Isup2.hkl

Additional supplementary materials:  crystallographic information; 3D view; checkCIF report

## Figures and Tables

**Fig. 1.**
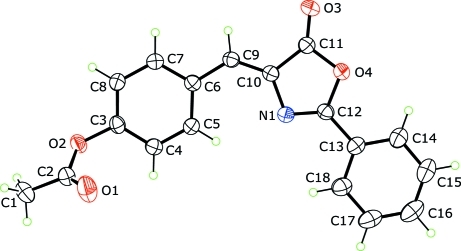
The molecular structure of (I) showing the atom-labelling scheme and displacement ellipsoids at the 35% probability level.

**Fig. 2.**
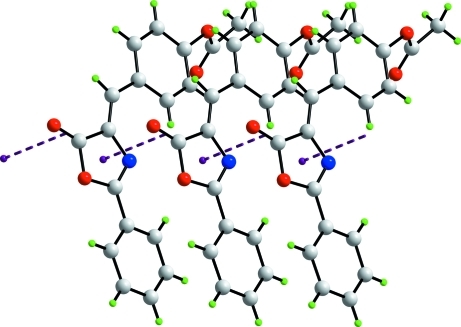
A supramolecular chain aligned along the b axis in (I), mediated by (C═ O)···π interactions (purple dashed lines). Colour code: O, red; N, blue; C, grey; and H, green.

## supplementary crystallographic information

### Comment

The 1,3-oxazole ring is known to have biological activity in its own right (Williams & Fu, 2010) and serves as a useful synthetic intermediate for the synthesis of imidazoles that are also possess a wide spectrum of biological activities, such as herbicides, fungicides, anti-bacterials, etc. (Khbnadidah *et al.*, 2003). In continuation of structural studies of oxazole compounds (Jotani & Baldaniya, 2008), the crystal structure of title compound, (I), is described herein.

The molecule of (I) is twisted around the C3–O2 bond as seen in the C2–O2–C3–C4 torsion angle of 58.2 (3) °. This results in a dihedral angle of 53.45 (7) ° between the acetyl residue [r.m.s. deviation = 0.0081 Å] and the remaining non-hydrogen atoms [r.m.s. = 0.0734 Å]; the dihedral angle formed between the two benzene rings is 5.10 (12) °. The configuration about the C9═C10 bond [1.343 (3) Å] is *Z*, and as the two benzene rings are orientated to the same side of the molecule, the overall molecular conformation is U-shaped. A similar conformation was reported in a di-methoxy derivative of (I), namely 2,6-dimethoxy-4-(5-oxo-2-phenyl-4,5-dihydro-1,3-oxazol-4-ylidenemethyl)- phenyl acetate (Sun *et al.*, 2007).

The crystal packing is dominated by (C═O)···π interactions that connect molecules into a linear supramolecular chain along the *b* axis, Fig. 2. The parameters defining this interaction are C11═O3···ring centroid(1,3-oxazole ring)^i^ = 3.370 (2) Å and angle = 85.11 (14) ° for *i*: *x*, 1+*y*, *z*.

### Experimental

A mixture of 4-acetoxyoxy benzaldehyde (0.25 mol), benzoyl amino acetic acid (0.25 mol), acetyl acetate (0.30 mol) and anhydrous sodium acetate (0.25 mol) were taken in a 500 ml round bottom flask and heated on an electric hot plate with constant stirring. After the complete liquefaction of the mixture, the flask was transferred to a sand bath and further heated for 2.5 h. Ethanol (100 ml) was added slowly to the flask and the mixture was allowed to stand overnight. The crystalline product obtained was filtered with ice-cold alcohol and then with boiling water. The crude product was crystallised from ethanol (95%) to obtain the final product (78% yield; m.pt. 428 K). The colourless crystals were obtained by slow evaporation from an ethanol solution of (I).

### Refinement

The H atoms were geometrically placed (C–H = 0.93–0.96 Å) and refined as riding with *U_iso_*(H) = 1.2–1.5*U_eq_*(C).

### Crystal data

**Table:**

C_18_H_13_NO_4_	*F*(000) = 640
*M**_r_* = 307.29	*D*_x_ = 1.372 Mg m^−^^3^
Monoclinic, *P*2_1_/*c*	Cu *K*α radiation, λ = 1.54180 Å
Hall symbol: -P 2ybc	Cell parameters from 25 reflections
*a* = 13.3507 (15) Å	θ = 20.0–30.0°
*b* = 3.9443 (9) Å	µ = 0.81 mm^−^^1^
*c* = 28.527 (5) Å	*T* = 293 K
β = 98.025 (11)°	Block, colourless
*V* = 1487.5 (5) Å^3^	0.40 × 0.20 × 0.15 mm
*Z* = 4	

### Data collection

**Table:**

Enraf–Nonius CAD-4 diffractometer	1795 reflections with *I* > 2σ(*I*)
Radiation source: fine-focus sealed tube	*R*_int_ = 0.054
graphite	θ_max_ = 64.9°, θ_min_ = 3.1°
2θ scan	*h* = 0→15
Absorption correction: ψ scan (North *et al.*, 1968)	*k* = 0→4
*T*_min_ = 0.852, *T*_max_ = 0.997	*l* = −33→33
2593 measured reflections	2 standard reflections every 3600 min
2491 independent reflections	intensity decay: none

### Refinement

**Table:**

Refinement on *F*^2^	Secondary atom site location: difference Fourier map
Least-squares matrix: full	Hydrogen site location: inferred from neighbouring sites
*R*[*F*^2^ > 2σ(*F*^2^)] = 0.050	H-atom parameters constrained
*wR*(*F*^2^) = 0.140	*w* = 1/[σ^2^(*F*_o_^2^) + (0.0834*P*)^2^ + 0.1813*P*] where *P* = (*F*_o_^2^ + 2*F*_c_^2^)/3
*S* = 1.06	(Δ/σ)_max_ < 0.001
2491 reflections	Δρ_max_ = 0.23 e Å^−^^3^
210 parameters	Δρ_min_ = −0.23 e Å^−^^3^
0 restraints	Extinction correction: *SHELXL97* (Sheldrick, 2008), Fc^*^=kFc[1+0.001xFc^2^λ^3^/sin(2θ)]^-1/4^
Primary atom site location: structure-invariant direct methods	Extinction coefficient: 0.0081 (8)

### Special details

**Table:**

Geometry. All s.u.'s (except the s.u. in the dihedral angle between two l.s. planes) are estimated using the full covariance matrix. The cell s.u.'s are taken into account individually in the estimation of s.u.'s in distances, angles and torsion angles; correlations between s.u.'s in cell parameters are only used when they are defined by crystal symmetry. An approximate (isotropic) treatment of cell s.u.'s is used for estimating s.u.'s involving l.s. planes.
Refinement. Refinement of *F*^2^ against ALL reflections. The weighted *R*-factor wR and goodness of fit *S* are based on *F*^2^, conventional *R*-factors *R* are based on *F*, with *F* set to zero for negative *F*^2^. The threshold expression of *F*^2^ > 2σ(*F*^2^) is used only for calculating *R*-factors(gt) etc. and is not relevant to the choice of reflections for refinement. *R*-factors based on *F*^2^ are statistically about twice as large as those based on *F*, and *R*- factors based on ALL data will be even larger.

### Fractional atomic coordinates and isotropic or equivalent isotropic displacement parameters (Å^2^)

**Table:**

	*x*	*y*	*z*	*U*_iso_*/*U*_eq_	
O1	0.98210 (13)	−0.0744 (6)	0.36427 (7)	0.0805 (7)	
O2	0.96858 (10)	−0.3277 (4)	0.43327 (5)	0.0501 (4)	
O3	0.36614 (12)	0.4187 (5)	0.44993 (6)	0.0606 (5)	
O4	0.32332 (10)	0.1834 (4)	0.37774 (5)	0.0466 (4)	
N1	0.47109 (13)	−0.0534 (5)	0.36494 (6)	0.0430 (5)	
C1	1.12156 (18)	−0.3904 (7)	0.40327 (10)	0.0628 (7)	
H1A	1.1314	−0.5250	0.3763	0.094*	
H1B	1.1304	−0.5297	0.4311	0.094*	
H1C	1.1700	−0.2091	0.4069	0.094*	
C2	1.01799 (17)	−0.2475 (7)	0.39635 (9)	0.0496 (6)	
C3	0.86835 (15)	−0.2184 (6)	0.43289 (8)	0.0425 (5)	
C4	0.79303 (16)	−0.3064 (6)	0.39696 (8)	0.0447 (6)	
H4	0.8082	−0.4318	0.3713	0.054*	
C5	0.69517 (15)	−0.2074 (6)	0.39935 (7)	0.0414 (5)	
H5	0.6442	−0.2660	0.3751	0.050*	
C6	0.67139 (15)	−0.0187 (6)	0.43805 (7)	0.0388 (5)	
C7	0.74930 (16)	0.0581 (6)	0.47402 (8)	0.0453 (6)	
H7	0.7348	0.1787	0.5003	0.054*	
C8	0.84763 (16)	−0.0399 (6)	0.47180 (7)	0.0479 (6)	
H8	0.8990	0.0138	0.4962	0.057*	
C9	0.57088 (15)	0.1106 (6)	0.44158 (7)	0.0406 (5)	
H9	0.5640	0.2187	0.4699	0.049*	
C10	0.48645 (15)	0.0971 (6)	0.40999 (7)	0.0396 (5)	
C11	0.39108 (16)	0.2560 (6)	0.41824 (8)	0.0435 (5)	
C12	0.37851 (15)	0.0018 (6)	0.34861 (7)	0.0410 (5)	
C13	0.32449 (17)	−0.1052 (6)	0.30296 (8)	0.0449 (6)	
C14	0.22131 (19)	−0.0575 (7)	0.29152 (9)	0.0566 (7)	
H14	0.1849	0.0484	0.3129	0.068*	
C15	0.1725 (2)	−0.1672 (8)	0.24835 (10)	0.0686 (8)	
H15	0.1030	−0.1381	0.2409	0.082*	
C16	0.2258 (2)	−0.3186 (7)	0.21650 (9)	0.0692 (8)	
H16	0.1924	−0.3929	0.1875	0.083*	
C17	0.3283 (2)	−0.3612 (7)	0.22719 (9)	0.0673 (8)	
H17	0.3644	−0.4603	0.2051	0.081*	
C18	0.3783 (2)	−0.2584 (7)	0.27036 (8)	0.0567 (7)	
H18	0.4477	−0.2913	0.2777	0.068*	

### Atomic displacement parameters (Å^2^)

**Table:**

	*U*^11^	*U*^22^	*U*^33^	*U*^12^	*U*^13^	*U*^23^
O1	0.0539 (11)	0.1108 (18)	0.0780 (13)	0.0078 (11)	0.0130 (9)	0.0395 (13)
O2	0.0390 (8)	0.0609 (11)	0.0510 (9)	0.0101 (7)	0.0086 (7)	0.0066 (8)
O3	0.0503 (10)	0.0774 (13)	0.0557 (10)	0.0091 (9)	0.0136 (8)	−0.0205 (10)
O4	0.0387 (8)	0.0522 (10)	0.0487 (9)	0.0046 (7)	0.0056 (6)	−0.0073 (8)
N1	0.0432 (10)	0.0431 (11)	0.0428 (10)	0.0031 (9)	0.0060 (8)	−0.0049 (9)
C1	0.0478 (14)	0.0644 (18)	0.0793 (17)	0.0082 (13)	0.0195 (12)	−0.0008 (15)
C2	0.0418 (12)	0.0538 (16)	0.0533 (13)	−0.0003 (11)	0.0075 (10)	−0.0007 (12)
C3	0.0369 (11)	0.0450 (13)	0.0460 (12)	0.0036 (10)	0.0069 (9)	0.0089 (11)
C4	0.0455 (12)	0.0452 (14)	0.0442 (12)	0.0020 (11)	0.0088 (9)	−0.0014 (11)
C5	0.0412 (11)	0.0422 (13)	0.0401 (11)	−0.0019 (10)	0.0034 (9)	−0.0006 (10)
C6	0.0407 (11)	0.0385 (13)	0.0380 (10)	−0.0005 (9)	0.0085 (9)	0.0045 (10)
C7	0.0442 (12)	0.0521 (14)	0.0400 (11)	0.0002 (11)	0.0070 (9)	−0.0052 (11)
C8	0.0397 (12)	0.0605 (16)	0.0423 (12)	−0.0009 (11)	0.0017 (9)	−0.0023 (11)
C9	0.0419 (11)	0.0411 (13)	0.0400 (11)	−0.0015 (10)	0.0096 (9)	−0.0022 (10)
C10	0.0402 (11)	0.0386 (13)	0.0410 (11)	0.0010 (10)	0.0088 (9)	−0.0017 (10)
C11	0.0410 (11)	0.0472 (14)	0.0430 (11)	−0.0009 (10)	0.0078 (9)	−0.0021 (11)
C12	0.0420 (12)	0.0377 (12)	0.0441 (11)	0.0013 (10)	0.0088 (9)	−0.0023 (10)
C13	0.0534 (13)	0.0385 (13)	0.0414 (11)	−0.0013 (10)	0.0019 (9)	0.0027 (10)
C14	0.0571 (15)	0.0563 (16)	0.0531 (14)	0.0004 (12)	−0.0033 (11)	0.0013 (13)
C15	0.0656 (16)	0.0642 (19)	0.0689 (17)	−0.0058 (15)	−0.0158 (14)	0.0039 (15)
C16	0.100 (2)	0.0493 (17)	0.0513 (15)	−0.0104 (16)	−0.0120 (15)	−0.0002 (13)
C17	0.095 (2)	0.0578 (18)	0.0476 (14)	0.0008 (15)	0.0035 (14)	−0.0093 (13)
C18	0.0656 (16)	0.0528 (16)	0.0512 (14)	0.0007 (13)	0.0065 (11)	−0.0057 (12)

### Geometric parameters (Å, °)

**Table:**

O1—C2	1.188 (3)	C6—C9	1.452 (3)
O2—C2	1.356 (3)	C7—C8	1.378 (3)
O2—C3	1.404 (2)	C7—H7	0.9300
O3—C11	1.193 (3)	C8—H8	0.9300
O4—C12	1.385 (2)	C9—C10	1.343 (3)
O4—C11	1.394 (3)	C9—H9	0.9300
N1—C12	1.277 (3)	C10—C11	1.468 (3)
N1—C10	1.404 (3)	C12—C13	1.460 (3)
C1—C2	1.481 (3)	C13—C14	1.384 (3)
C1—H1A	0.9600	C13—C18	1.390 (3)
C1—H1B	0.9600	C14—C15	1.380 (4)
C1—H1C	0.9600	C14—H14	0.9300
C3—C8	1.375 (3)	C15—C16	1.367 (4)
C3—C4	1.376 (3)	C15—H15	0.9300
C4—C5	1.374 (3)	C16—C17	1.371 (4)
C4—H4	0.9300	C16—H16	0.9300
C5—C6	1.404 (3)	C17—C18	1.377 (3)
C5—H5	0.9300	C17—H17	0.9300
C6—C7	1.389 (3)	C18—H18	0.9300
			
C2—O2—C3	119.26 (17)	C10—C9—C6	129.6 (2)
C12—O4—C11	105.40 (16)	C10—C9—H9	115.2
C12—N1—C10	105.77 (17)	C6—C9—H9	115.2
C2—C1—H1A	109.5	C9—C10—N1	129.26 (19)
C2—C1—H1B	109.5	C9—C10—C11	122.8 (2)
H1A—C1—H1B	109.5	N1—C10—C11	107.96 (18)
C2—C1—H1C	109.5	O3—C11—O4	121.40 (19)
H1A—C1—H1C	109.5	O3—C11—C10	133.7 (2)
H1B—C1—H1C	109.5	O4—C11—C10	104.85 (18)
O1—C2—O2	123.0 (2)	N1—C12—O4	115.99 (18)
O1—C2—C1	126.2 (2)	N1—C12—C13	127.46 (19)
O2—C2—C1	110.7 (2)	O4—C12—C13	116.54 (18)
C8—C3—C4	121.5 (2)	C14—C13—C18	119.5 (2)
C8—C3—O2	116.73 (19)	C14—C13—C12	121.5 (2)
C4—C3—O2	121.6 (2)	C18—C13—C12	119.0 (2)
C5—C4—C3	119.5 (2)	C15—C14—C13	119.9 (3)
C5—C4—H4	120.3	C15—C14—H14	120.1
C3—C4—H4	120.3	C13—C14—H14	120.1
C4—C5—C6	120.7 (2)	C16—C15—C14	120.3 (3)
C4—C5—H5	119.7	C16—C15—H15	119.8
C6—C5—H5	119.7	C14—C15—H15	119.8
C7—C6—C5	117.93 (19)	C15—C16—C17	120.1 (2)
C7—C6—C9	118.43 (19)	C15—C16—H16	119.9
C5—C6—C9	123.59 (19)	C17—C16—H16	119.9
C8—C7—C6	121.6 (2)	C16—C17—C18	120.5 (3)
C8—C7—H7	119.2	C16—C17—H17	119.8
C6—C7—H7	119.2	C18—C17—H17	119.8
C3—C8—C7	118.7 (2)	C17—C18—C13	119.7 (3)
C3—C8—H8	120.6	C17—C18—H18	120.2
C7—C8—H8	120.6	C13—C18—H18	120.2
